# MR-guided cardiac radiofrequency ablation with catheter-tracked local MR lesion monitoring

**DOI:** 10.1186/1532-429X-15-S1-M10

**Published:** 2013-01-30

**Authors:** Steffen Weiss, Sascha Krueger, Peter Koken, Gregg Stenzel, Lars Bloch, Won Yong Kim, Anne Yoon Krogh Grøndal, James Harrison, Mark O'Neill, Reza Razavi, Tobias Schaeffter

**Affiliations:** 1 Division of Imaging Sciences and Biomed Eng., King's College London, London, UK; 2 Research Laboratories, Philips Technologie GmbH, Hamburg, Germany; 3 Department of Cardiology, Aarhus University, Skeyby Hospital, Aarhus, Denmark; 4 Imricor Medical Systems, Inc., Burnsville, MN, USA

## Background

MR-guided electrophysiology (MR-EP) for the treatment of arrhythmia has the potential advantages of improved soft tissue contrast navigation and visualization of ablation-induced tissue changes. In the past, the development of MR-conditional EP catheters and an appropriate image guidance platform have represented major obstacles. The aim of this work was to establish and evaluate a catheter-guided approach that allows monitoring of radiofrequency (RF) ablation at the electrode tip.

## Methods

**Figure 1:**
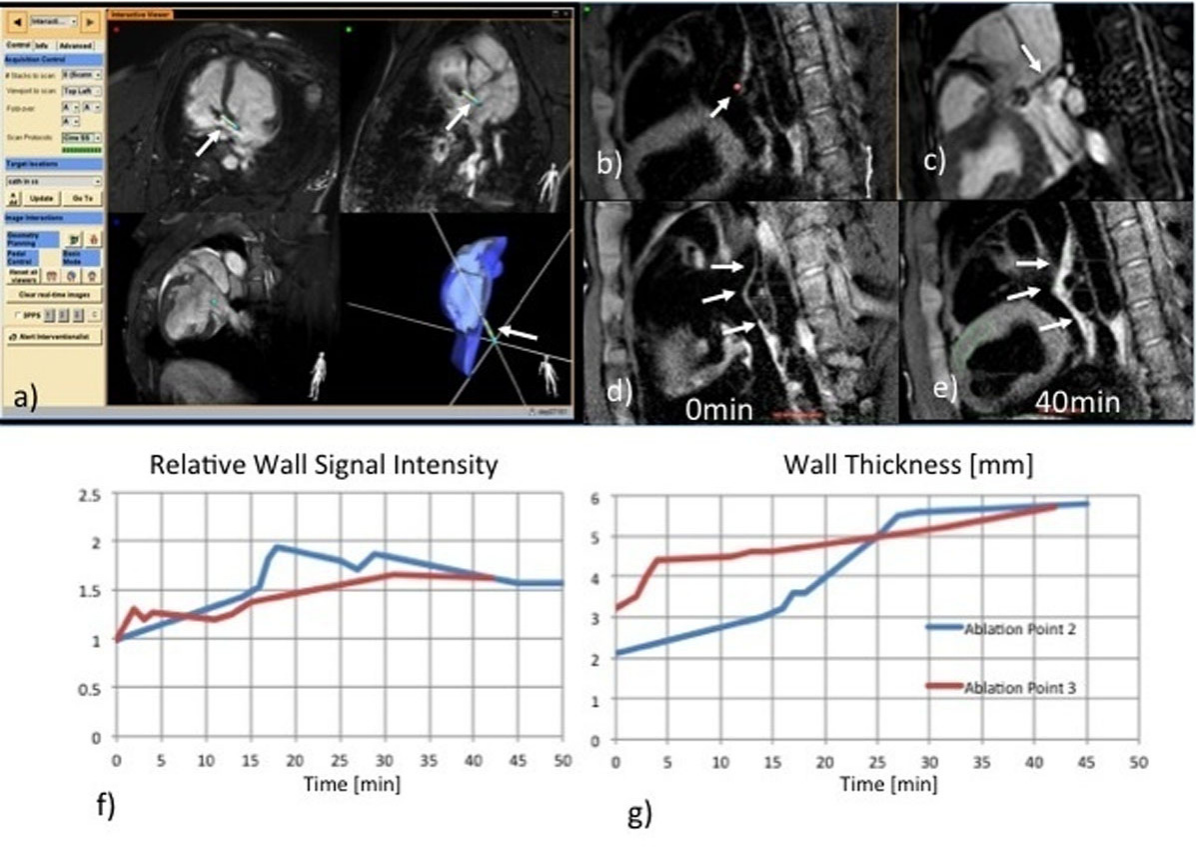
Catheter tip is displayed relative to multi-planar reformatted slices and a surface-rendered cardiac model (a). The catheter tip position obtained from tracking (b) and verified visually in a SSFP cine scan (c). Wall thickness and T2-signal of SVC, IVC before and 40 min after ablation (d,e). Wall thickness and T2-signal enhancement over time (f,g)

A deflectable ablation catheter (Vision MRM, Imricor, Burnsville, MN) with two RF micro-coils allows tracking of the catheter tip and its orientation. The integration of dedicated transmission line technology ensures safe tracking. Continuous acquisition of projections allows tracking of the catheter motion with an update rate of 10Hz. A prototype platform (MR iSuite) was used to display the position and the orientation of the catheter tip relative to multi-planar reformatted or real-time slices and to a surface-rendered cardiac model in 3D (Figure 1a) for improved catheter guidance. The iSuite allows automatic planning of MR scans relative to the position and orientation of the catheter. This mechanism was used to invoke the acquisition of two different image datasets on a 1.5T MR-scanner (Achieva, Philips Healthcare, Netherlands) to prepare and monitor RF ablation. First, a cardiac-gated SSFP cine sequence (TR/TE=3.2/1.6ms, 40 phases, resolution: 1.4x1.4x8mm, AQ=15sec) is obtained to verify sufficient contact of the catheter tip to the tissue. Secondly, a high-resolution cardiac-triggered black blood T2-weighted TSE sequence (TE=50ms, Turbo-Factor=16, resolution: 0.95x0.95x4mm, AQ=12sec) is used to visualize the effect of the RF ablation on the tissue (edema and wall-thickness).

This approach was first tested in a phantom and then assessed in-vivo in a 40kg pig. Four discrete RF lesions (40W, 1min) were applied on the posterior right atrial wall. Edema and wall thickness were monitored for each catheter tip location over the first 5min and at 15, 30, and 45min. The T2-signal in the wall was normalized to the ventricular myocardial signal to determine a relative enhancement.

## Results

Catheter tracking at 10Hz was reliable for all catheter tip orientations, resulting in smooth catheter guidance and accurate automatic scan plane planning.

The in-vivo results are shown in Figure 1. The position of the catheter tip (Figure 1b) is confirmed by cardiac cine SSFP imaging (Figure 1c). The images for measurement of the wall thickness and the signal enhancement are shown in Figure 1d+e, before and 30min after ablation. The wall thickness and T2-signal enhancement at two ablation sites increase to approx. twice the original value during the first 30min (Figure 1f+g).

## Conclusions

A new technique to quantify wall thickness and T2-signal enhancement at the catheter tip has been demonstrated. This allows for fast and efficient monitoring of catheter-based RF ablation.

## Funding

This work has been funded by the Techology Strategy Board (TS/G02142/1) and the Wellcome/EPSRC Medical Engineering Centre.

